# Cultural adaptation, reliability and validity of Japanese Orthopaedic Association Back Pain Evaluation Questionnaire to Brazilian Portuguese

**DOI:** 10.1590/S1679-45082017AO3890

**Published:** 2017

**Authors:** Patrícia Rios Poletto, Daiana Kerry Picanço Gobbo, Alberto Ofenhejm Gotfryd, Sabrina Nieto Catania, Daphine de Carvalho Sousa, Sarita Barbosa Sanches Pereira

**Affiliations:** 1 Universidade Federal de São Paulo, Santos, SP, Brazil.; 2 Santa Casa de Misericórdia de São Paulo, São Paulo, SP, Brazil.; 3 Hospital Israelita Albert Einstein, São Paulo, SP, Brazil.; 4 Santa Casa de Misericórdia de Santos, Santos, SP, Brazil.

**Keywords:** Low back pain, Surveys and questionnaires, Translating, Validation studies, Dor lombar, Inquéritos e questionários, Tradução, Estudos de validação

## Abstract

**Objective:**

To describe the translation and cultural adaptation of the Japanese Orthopaedic Association Back Pain Evaluation Questionnaire into Brazilian Portuguese, and verifies the reliability and validity of this new version.

**Methods:**

A cross-cultural adaptation of the Japanese Orthopaedic Association Back Pain Evaluation Questionnaire was performed using the following steps: translation, back-translation, committee review, and pre-testing phase (50 subjects). The psychometric properties were evaluated by application of the questionnaire to 102 patients. Reliability was assessed by homogeneity and stability of measures. The criterion-related validity was tested by comparing scores of Japanese Orthopaedic Association Back Pain Evaluation Questionnaire to Oswestry and Medical Outcomes Study 36 - Item Short questionnaires.

**Results:**

Excellent internal consistency was found in both test (Cronbach’s α of 0.90) and re-test (Cronbach’s α of 0.91). The Japanese Orthopaedic Association Back Pain Evaluation Questionnaire showed good reliability and the correlations ranged from reasonable (0.64) to very good (r=0.91).

**Conclusion:**

The Brazilian Portuguese version of Medical Outcomes Study 36 - Item Short was easy to apply and understand. The questionnaire had a great impact on assessment and multidimensional care of patients with low back pain.

## RESUMO

**Objetivo:**

Realizar a tradução e a adaptação cultural para a língua portuguesa do Brasil do Japanese Orthopaedic Association Back Pain Evaluation Questionnaire para avaliação de dor lombar, e verificar a confiabilidade e a validade da nova versão.

**Métodos:**

A adaptação cultural foi realizada de acordo com as seguintes etapas: tradução, retrotradução, revisão por comitê e pré-teste (50 indivíduos). Após, as propriedades psicométricas foram avaliadas aplicando-se o questionário (teste) em 102 pacientes. A confiabilidade foi verificada por avaliação da homogeneidade e da estabilidade das medidas. A validade de critério foi testada comparando-se as pontuações do Japanese Orthopaedic Association Back Pain Evaluation Questionnaire com os questionários Oswestry e Medical Outcomes Study 36 - Item Short.

**Resultados:**

Observou-se excelente consistência interna no pré-teste (Cronbach α de 0,90) e no teste (Cronbach α de 0,91). O Japanese Orthopaedic Association Back Pain Evaluation Questionnaire apresentou boa confiabilidade, e as correlações variaram de razoável (0,64) a muito boa (r=0,91).

**Conclusão:**

A versão em língua portuguesa do Brasil é de fácil aplicação e compreensão, além de apresentar grande acréscimo na avaliação e no cuidado multidimensional de pacientes portadores de dor lombar.

## INTRODUCTION

Low back pain is a common musculoskeletal disorder that affects mainly economically active adults.^(^ ^1^ ^)^ This disease is an important factor that causes absences from work, therefore, massive economic and social impact.^(^ ^1^ ^,^ ^2^ ^)^ Strategies to treat chronic low back pain vary considerably from country to country or even in different areas within the same country.

The prognosis of chronic low back pain is uncertain because it includes several interlinked variables, which stimulates the search for new approaches to deal with this problem.^(^ ^3^ ^)^ Therefore, general aspects of low back pain have been studied, including disability, rehabilitation, and compliance.^(^ ^4^ ^,^ ^5^ ^)^ Several reports described different scales and questionnaires to assess a number of aspects of low back pain, including perceived disability, quality of life, severity, intensity, pain distribution, and functional status.^(^ ^6^ ^-^ ^8^ ^)^

The number of publications on lumbar degenerative disorders has significantly increased in Asian countries in recent decades. In many studies, Japanese Orthopaedic Association Back Pain Evaluation Questionnaire (JOABPEQ) published a review of this questionnaire that had improved outcomes assessment. After, several studies showed the efficacy of JOABPEQ to evaluate lumbar disorders.^(^ ^9^ ^-^ ^12^ ^)^ It is a simple and effective method to assess lumbar back pain. In addition, the need of comparing different ethnic groups motivated the development of a Brazilian version of the questionnaire.

## OBJECTIVE

To describe the translation and cultural adaptation of the Japanese Orthopaedic Association Back Pain Evaluation Questionnaire into Brazilian Portuguese, and verifies the reliability and validity of this new version.

## METHODS

### Subjects

The JOABPEQ was applied in patients from the outpatient clinic of orthopedic diseases of the *Santa Casa de Misericórdia de Santos* between 2011 and 2013. All patients received explanation about the study and those who agreed to participate signed the Informed Consent. The study was approved by the Institutional Ethics and Research Committee, with CAAE: 21338713.3.0000.5478/670.821.

In the pre-test evaluation we included 50 subjects (62% women; 49.4±8.79 years old) to apply the preliminary version of the questionnaire. In the second step, 102 adults were included (65.7% women; 47.9±9.17 years old) in the main study and they responded to the translated version of JOABPEQ.

The inclusion criteria were presence of chronic lumbar back pain (minimum 7 weeks of symptoms) and patients aged 18 and 70 years. In order to homogenize the sample, we excluded all patients who underwent surgery. Patients were not classified by disease or type of treatment. We did not considered any correlation between nosological classification of diseases and JOABPEQ scores, because it was not the goal of our study.

All the patients were selected from the Orthopaedic and Physical Therapy Departments of *Santa Casa de Misericórdia de Santos* School Hospital. Participants completed forms during their rehabilitation period. Patients’ clinical history were evaluated to verify if they met the inclusion criteria of the study, and those who fulfilled the criteria were invited to participate during their clinical follow-up visits.

### Instruments

The JOABPEQ has 25 questions that analysis and interpretation were grouped in the following sub-scales: low back pain, lumbar function, walk ability, social life function and mental health. The higher the score the better the patient’s condition. The end of the questionnaire presents three visual scales so we can complete the assessment in relation to pain with degrees for low back pain, buttocks and lower limb pain, and numbness in buttocks and lower limb (zero for comfortable condition without any pain at all to 10 for the most intense pain/numbness imaginable).

For the process of translation, cultural adaptation and validation of the JOABPEQ in the Brazilian population, we also used the Brazilian versions of Oswestry^(^ ^13^ ^)^ and Medical Outcomes Study 36 - Item Short (SF-36)^(^ ^14^ ^)^ questionnaires.

The Brazilian version of Oswestry^(^ ^13^ ^)^ allow the evaluation of functional disability presented by the patient, based on the level of pain, at different activities in daily life. This questionnaire includes ten questions about daily life activities and patient’s responses to the questions assessing the pain impact during these activities. Results range from zero (minimum disability) to 100 (disability).

The SF-36 questionnaires,^(^ ^14^ ^)^ also validated for Brazilian population, was used to evaluate quality of life, through 8 areas divided into 12 questions. The impact of questions is measured according to the authors’ recommendation to classify patients. Results are given separately for each domain, ranging from zero to 100, and 100 is the best possible result.

### Proceedings

The translation of the JOABPEQ into Brazilian Portuguese followed the recommendations of American Association of Orthopedic Surgery (AAOS) and the International Quality of Life Assessment (IQOLA) guidelines,^(^ ^15^ ^-^ ^17^ ^)^ according to the following steps: the original English questionnaire was translated into Portuguese by two independent translators, both fluent in the target language with good understanding of source language (blinded translation); synthesis of the two Portuguese versions in a single version was done by a trained professional in languages who checked spelling, grammar and overall style and response options; retranslation into English was done by two independent translators who were fluent in the source language (blinded retranslation); synthesis of the two English versions was compiled in a single version by a native English speaker language professional, and this new version was compared to the original version of the questionnaire for consistence; review by committee composed by health professionals, English and Portuguese speaking professionals and members who participated in the questionnaire preliminary analysis, and its main function was to verify the interpretation of each question and the answers; pre-test application for individuals who had low back pain. This phase was important to identify the understanding, acceptance and emotional impact of the questionnaire items, in addition to detect items that were confuse or misunderstanding. After that, a consensus meeting was promoted to the needed minor semantic adjustments to final edition of the questionnaire new version (Appendix 1). At least, the main part of the study included an exploratory and confirmatory analysis to determine the JOABPEQ psychometric properties (reliability and validity). Reliability was assessed using stability (test-retest interval of 4 days between assessments) and analysis of internal consistency. One hundred and two adults participated in this final this step.

The validity of JOABPEQ was determined by the assessment of criterion-related validity, which verifies the association between the instrument with others questionnaires that are recognized as valid. The questionnaires we used were already described above.

The retest step was possible because patients who were part of the study were monitored periodically by the orthopedic diseases clinic of the *Santa Casa de Misericórdia de Santos* and their data were strictly recorded to allow monitoring during the period of the study.

### Statistical analysis

To statistical analysis we used the Statistic Package Social Science (SPSS) version 17. Internal consistency was checked with Cronbach’s α coefficient for JOABPEQ results in test and retest. The test-retest reliability was assessed using Pearson’s correlation coefficient with paired *t*-test. The evaluation of the validity (criterion-related validity) was performed using Pearson’s correlation coefficient, ranging from 0.00 to 1.00 (0.00 to 0.25 indicated poor association; 0.26 to 0.49, low association; 0.50 to 0.69, moderate association; 0.70 to 0.89, high association; and 0.90 to 1.00, very high association). The level of significance adopted was 5%.

## RESULTS

The cultural adaptation process occurred successfully, and all steps were followed. The questionnaire was applied in pre-test phase among fifty patients. After this step, we revised the questionnaire for data collection and statistical analysis. Reliability was assessed by internal consistency and stability (test-retest). Regarding internal consistency analysis, JOABPEQ showed excellent results in both test (Cronbach’s α of 0.90) and retest (Cronbach’s α of 0.91). The table 1 shows results from the analysis of stable measurements.

**Table 1:** Stability of the Japanese Orthopaedic Association Back Pain Evaluation Questionnaire

JOABPEQ	Pearson’s correlation coefficient			Paired *t* test		
	
	r	p value	Association power	Mean difference	p value	Concordance
Lumbar pain	0.64	0.000*	Moderate	2.38	0.220	Yes
Lumbar function	0.88	0.000*	Very high	3.55	0.026*	No
Deambulation	0.91	0.000*	Very high	0.69	0.602	Yes
Social life	0.89	0.000*	Very high	1.46	0.223	Yes
Mental health	0.91	0.000*	Very high	1.71	0.049*	No

* Statistical differences between groups (p<0.005).

JOABPEQ: Japanese Orthopaedic Association Back Pain Evaluation Questionnaire.

Although high degree of association was seen between the results in the test and retest (Pearson’s correlation coefficient results), the mean difference between this score (paired *t*-test results) showed that the agreement is weak for the lumbar function and mental health domains, which shows a weak reliability for these items. In the areas of lumbar pain, ambulation and social life, good reliability was found.

The validity of JOAPEQ was measured of association with the other instruments already validated for the Portuguese language (results of Pearson’s correlation coefficient). These results can be seen in tables 2 and 3.

**Table 2:** Results of association between Japanese Orthopaedic Association Back Pain Evaluation Questionnaire and Oswestry

JOABPEQ	Oswestry	

	r	p value
Lumbar pain	-0.33	0.001*
Lumbar function	-0.62	0.000*
Deambulation	-0.66	0.000*
Social life	-0.78	0.000*
Mental health	-0.58	0.000*

* Statistical differences between groups (p<0.005).

JOABPEQ: Japanese Orthopaedic Association Back Pain Evaluation Questionnaire.

**Table 3:** Association between Japanese Orthopaedic Association Back Pain Evaluation Questionnaire and Medical Outcomes Study 36 - Item Short (SF-36)

SF-36	JOABPEQ									

	Lumbar pain		Lumbar function		Deambulation		Social life		Mental health	
				
	r	p value	r	p value	r	p value	r	p value	r	p value
Functional capacity	0.41	0.000*	0.70	0.000*	0.79	0.000*	0.79	0.000*	0.60	0.000*
Physical limitation	0.26	0.000*	0.42	0.000*	0.41	0.000*	0.59	0.000*	0.43	0.000*
Pain	0.39	0.000*	0.47	0.000*	0.49	0.000*	0.76	0.000*	0.59	0.000*
General health	0.25	0.012*	0.42	0.000*	0.38	0.000*	0.43	0.000*	0.58	0.000*
Vitality	0.26	0.009*	0.40	0.000*	0.32	0.001*	0.49	0.000*	0.74	0.000*
Social aspects	0.31	0.002*	0.28	0.004*	0.34	0.000*	0.54	0.000*	0.70	0.000*
Emotional aspects	0.24	0.016*	0.24	0.015*	0.28	0.004*	0.43	0.000*	0.48	0.000*
Mental health	0.23	0.018*	0.22	0.028*	0.13	0.193	0.36	0.000*	0.73	0.000*

* Statistical differences between groups (p<0.005)

JOABPEQ: Japanese Orthopaedic Association Back Pain Evaluation Questionnaire.

The association between results of JOAPEQ and the Oswestry questionnaire was statistically significant. In table 2, we can verify a high association of Oswestry with JOABPEQ in the social life domain. The association with Oswestry and the JOABPEQ domain lumbar function, deambulation and mental health was moderate. And finally, the association with Oswestry and the JOABPEQ domain lumbar pain was low. Results of association were negative because, for JOAPBEQ, the best condition of the patient represented the highest values in the questionnaire, and, for Oswestry, the best condition of the patient was identified by the lower values in the questionnaire.

Table 3 shows results of association between JOABPEQ and SF-36. The results of the association were positive because, for JOAPBEQ and SF-36, the best condition of the patient represented the highest values.

Association was statistically significant between questionnaires in all domains, except for JOABPEQ deambulation and SF-36 mental health. JOABPEQ mental health had high association with SF-36 vitality, social functioning, and mental health, and moderate association with functional capacity, pain and general health status. JOABPEQ social life had the best association with SF-36 functional capacity and pain. There was a high association between JOABPEQ deambulation and lumbar function in SF-36 functional capacity.

## DISCUSSION

The JOABPEQ is a questionnaire to assess various aspects of back pain, including perceived disability, quality of life, severity, intensity, pain distribution and functional status.

This study described the translation and cultural validation into Brazilian Portuguese of the JOABPEQ. The JOABPEQ is a relatively new assessment tool, which was validated in Japan in 2007 by Fukui et al.^(^ ^10^ ^)^ The assessment tool score includes five categories (25 items) selected from the Roland Morris Disability Questionnaire, SF-36, and a Visual Analogue Scale. The results range from zero (worst pain) to 100 (no pain).

Recently, the number of Asian publications has increased. This, added to the fact that the JOABPEQ is frequently cited in a number of studies, the translation into Brazilian Portuguese is relevant, particularly because it enables to compare treatments in different ethnic groups.

Several rating scales have been used for the analysis of patients with lumbar back pain. In Brazil, a Portuguese version of Oswestry questionnaire is often the scale of choice. For this reason we compared Oswestry questionnaire to the new Brazilian Portuguese version of JOABPEQ. In addition, two other tools used worldwide for analysis of quality of life (SF-36 and Visual Analogue Scale) were compared as valid measures.

The Brazilian Portuguese version of JOABPEQ had excellent internal consistency. In our study, the Cronbach’s α values observed on test and retest were 0.90 and 0.91, respectively. These values are better than the one described by Azimi et al., (0.71 and 0.81, respectively).^(^ ^18^ ^)^

Regarding results of measures of stability (test-retest), the Brazilian version of JOABPEQ had good reliability, excepting for the lumbar function and mental health domains. The hypothesis to explain might be that current version was applied exclusively to non-surgical patients, while the previous articles also evaluated surgical ones. Different diseases may lead to different scores results, and we have the influence of several factors on JOABPEQ results, such as sex, age and type of disease and treatment.^(^ ^12^ ^)^ It is also important to note that these domains may be influenced by other aspects of everyday life that could not be measured by the instrument.

Another factor that may have influenced these results was the time interval in test and retest. The interval between the test and retest assessment is an important factor to be considered.^(^ ^19^ ^)^ Long periods can compromise the interpretation of results because of clinical changes that may occur with the subject. On the other hand, too short times results can suffer from “memory effect” and do not represent reliable values. In our study, the average interval between evaluations was 4 days, ranging from 3 to 5, which is desirable in this kind of research.^(^ ^20^ ^)^

Another hypothesis to explain these discrepancies in these two domains may fall on the impact of statistical analysis chosen, as, of all the variables considered in the calculation, only one is ordinal, and the remaining nominal can lead to the divergent results found for these domains. This aspect should be considered as a limitation of this study.

To verify the validity of a questionnaire means to check whether it actually measures what it is intended to. For this evaluation, we analyzed the association of the JOABPEQ with other instruments already validated in the Portuguese language.

Most of the associations found between the JOABPEQ domains with the Oswestry and SF-36 questionnaires were moderate and high, which agreed and assured good use of the instrument in the Brazilian population.

The association of the JOABPEQ lumbar pain domain with the Oswestry questionnaire and all domains of the SF-36 questionnaire was considered low. This low association might occur because both Oswestry and SF-36 questionnaires do not directly assess pain intensity. In the Oswestry questionnaire only one of questions directly assessed the intensity of pain and the SF-36 questionnaire assessed pain through quality of life.

These variations may have occurred because of the type of patients included in our study. Our patients did not have degenerative changes of the lumbar spine and their clinical presentation had small changes that were not found equally in all instruments used during the validation process.

We have not found in the literature many validation studies of the JOABPEQ to other languages.^(^ ^18^ ^,^ ^19^ ^,^ ^21^ ^,^ ^22^ ^)^ In the existing publications, only Thailand’s version performed the JOABPEQ validation comparing with the SF-36 questionnaire and found results similar to our study.^(^ ^21^ ^)^

Another aspect that can be observed by comparing our study with the three others that translated JOABPEQ was that statistical tests chosen can developed non-comparable interpretations. Our study sought to follow international recommendations already mentioned, even concerning data analysis.

The qualitative advantage of the JOABPEQ questionnaire is that easiness and rapid application, to apply the questionnaire does not take longer than few minutes. Our study confirmed that no instrument applied alone is complete enough to evaluate a pain, mainly a complex pain such as chronic low back pain.

Limitations of this study were the difficulty some patients to respond the questionnaires because of their low educational level and the latent presence of chronic lumbar pain in part of the sample, a fact that may have influenced our results.

## CONCLUSION

The Japanese Orthopaedic Association Back Pain Evaluation Questionnaire adaptation process to the Brazilian Portuguese was adequate. The instrument had excellent psychometric properties and its application was reliable within Brazilian population.
